# Intervention-related, contextual and personal factors affecting the implementation of an evidence-based digital system for prevention and treatment of malnutrition in elderly institutionalized patients: a qualitative study

**DOI:** 10.1186/s12913-023-09227-8

**Published:** 2023-03-13

**Authors:** Cecilie Varsi, Lene Frost Andersen, Gunhild Tellebon Koksvik, Frida Severinsen, Mari Mohn Paulsen

**Affiliations:** 1 Faculty of Health and Social Sciences, University of South-Eastern Norway, box 4, Borre, 3199 Norway; 2 Department of Nutrition, Institute of Basic Medical Sciences, University of Oslo, box 1110, Blindern, Oslo, 0317 Norway

**Keywords:** Malnutrition, Elderly patient, The Consolidated Framework for implementation research, Decision support system, eHealth

## Abstract

**Background:**

Malnutrition in elderly institutionalized patients is a significant challenge associated with adverse health outcomes. The ‘MyFood’ decision support system was designed to prevent and treat malnutrition and has previously been studied in a hospital setting. The aim of this study was to explore the experiences of nursing staff regarding the implementation of MyFood in settings treating elderly patients.

**Methods:**

The study was conducted in two settings treating elderly patients in Norway. Nursing staff received training in how to follow-up patients with MyFood. Qualitative interviews were conducted with 12 nursing staff. The Consolidated Framework for Implementation Research (CFIR) was used to guide the data collection and the thematic data analysis.

**Results:**

The implementation of a digital decision support system to prevent and treat malnutrition into settings treating elderly patients was found to be affected by intervention-related, contextual, and personal factors. Although nursing staff experienced several advantages, the leadership engagement was low and hampered the implementation.

**Conclusion:**

Nursing staff experienced several advantages with implementing a digital decision support system for the prevention and treatment of malnutrition in institutionalized elderly patients, including quality improvements and time savings. The results indicate that the leadership engagement was weak and that some nursing staff experienced low self-efficacy in digital competence. Future improvements include increasing the level of training, using MyFood throughout the patient course and involving the patient’s next-of-kin.

**Trial registration:**

The study was acknowledged by The Norwegian Centre for Research Data (NSD), ref. number 135175.

## Background

Malnutrition in elderly institutionalized patients is underreported and undertreated [1] and recognized as a major challenge [2, 3]. Malnutrition is related to increased morbidity [4, 5], adverse outcomes [3, 6], impaired functional status [6], a longer length of hospital stay [7, 8], reduced quality of life [9] and increased risk of premature death [2, 7].

In Norway, the responsibility for nutritional care for patients in hospitals and nursing homes is shared between several health care providers. Nursing staff have a key-role in the prevention and treatment of malnutrition, including assessment of nutritional status, supervision, and monitoring of nutritional intake [10, 11]. Current treatment and follow-up of malnutrition have demonstrated to be insufficient and associated with several barriers, including poor routines for malnutrition screening and documentation of nutritional intake and treatment [12] and limited skills and knowledge on nutritional treatment among nursing staff [13]. Norwegian data indicate that at the most, only 50% of malnourished or at-risk patients receive nutritional treatment [14, 15], thus interventions for reducing malnutrition in elderly institutionalized patients are highly needed [2]. Two recent systematic reviews showed that in-hospital nutritional support was associated with improved survival, lower frequency of hospital-associated infections and lower rates of hospital readmissions, and that nutritional support is cost-effective [16, 17].

Digital tools and applications (apps) to assess malnutrition and monitor nutritional intake have shown to be effective in increasing malnutrition detection and awareness, and may also help to reduce health care providers’ workload and time spent assessing patients for malnutrition [18, 19].

In response to existing research and recommendations, the second (LFA) and last author (MMP) of the current research team have developed, evaluated, and tested the digital dietary assessment and decision support system ‘MyFood’ for a hospital setting in Norway. The proof-of-concept studies showed that the dietary recording functionality in MyFood was relatively accurate for patients suffering from hematological and gastrointestinal diseases [20]. MyFood was perceived as more trustworthy and motivational to use compared to current practice [21, 22], and use of the system led to a decrease in the proportion of patients at risk of malnutrition, compared to a control group [23]. MyFood is not only a digital system for dietary assessment, as it also aims to improve the health of patients by supporting and advising in dietary decisions. Thus, MyFood can be considered an intervention, in line with the definition of Smith et al.: “Any activity undertaken with the objective of improving human health by preventing disease, by curing or reducing the severity or duration of an existing disease, or by restoring function lost through disease or injury” [24].

Despite the promising effects of digital interventions for nutritional care obtained in studies [18], their implementation into clinical practice is less studied. However, studies on the implementation of nutritional care without digital components show that implementation can be demanding and hampered by several barriers such as lack of management support, lack of time, lack of knowledge and lack of motivation [25]. Systematic reviews have found that strong leadership, a supportive working environment and staff training programs are essential to effective nursing practice in supporting patients’ nutrition [2, 26]. Others have also highlighted the vital role of nurse managers in the implementation of evidence-informed practices, including providing a supportive culture and environment [27–33].

The previous studies of MyFood showed that several aspects affected the implementation, such as aspects related to the intervention itself (i.e., ease of use and trustworthiness), contextual factors (i.e., time and resources) and individual factors (i.e., compliance) [16, 17]. The natural next step in the current research portfolio is to test the implementation of MyFood in settings other than specialized hospital wards, and this article presents a study of MyFood in more generalized settings treating a diversity of elderly patients.

In the current study, the Consolidated Framework for Implementation Research (CFIR) [34] was used to explore aspects related to the implementation of MyFood. CFIR is a multi-dimensional implementation framework described as well suited to emphasize aspects related to the implementation of nutritional terminology and interventions [35, 36].

CFIR comprises 39 constructs sorted under five domains [34]:

1. Intervention characteristics (i.e., aspects related to the complexity, relative advantage, trialability, adaptability, quality and packaging of an intervention).
2. Outer setting (i.e., aspects related to the patients’ needs and resources and aspects related to policy, policymakers and peer pressure).
3. Inner setting (i.e., organizational structure, culture, implementation readiness, leadership engagement, communication and networks).
4. Characteristics of individuals (i.e., individual stage of change, knowledge of, belief in, and level of confidence in using the intervention).
5. Process (i.e., planning, execution, evaluation, and involvement of supportive resource persons).

This study aimed to explore the experiences of nursing staff regarding the implementation of an evidence-based digital system for the prevention and treatment of malnutrition in elderly institutionalized patients.

## Methods

### Study design and setting

This qualitative study reports on findings from individual interviews with nursing staff regarding their experiences of using MyFood, a digital decision support system for preventing and treating malnutrition. The study was conducted in two locations in Norway. The first location was a short-term nursing home department in a large municipality, where the study was conducted from August - November 2021. The second location was a geriatric and renal hospital department, where the study was conducted from May - June 2022. The selection of these specific locations was due to a request of testing the MyFood system from the registered dietitian in the municipality and from the department physician leader at the hospital.

### The MyFood system

MyFood is a digital dietary assessment and decision support system designed to prevent and treat disease-related malnutrition. The interface of MyFood includes an app for dietary recording and evaluation and a website with reports to health care providers for documentation. The web report also provides decision support for nutritional treatment and a draft for an individual nutrition care plan. The first prototype was developed and evaluated in the period from 2016 to 2019 [20, 23] and a revised version was developed in 2020–2021. The revised MyFood system included a larger variety of food and beverage items, the possibility to monitor nutritional intake over weeks and months, and the possibility for health care providers to record nutritional intake on behalf of the patients and to see an overview of all patients at their department simultaneously. A feature where the patient could read simple dietary advice depending on nutrition-related symptoms was also included.

**Fig. 1:**
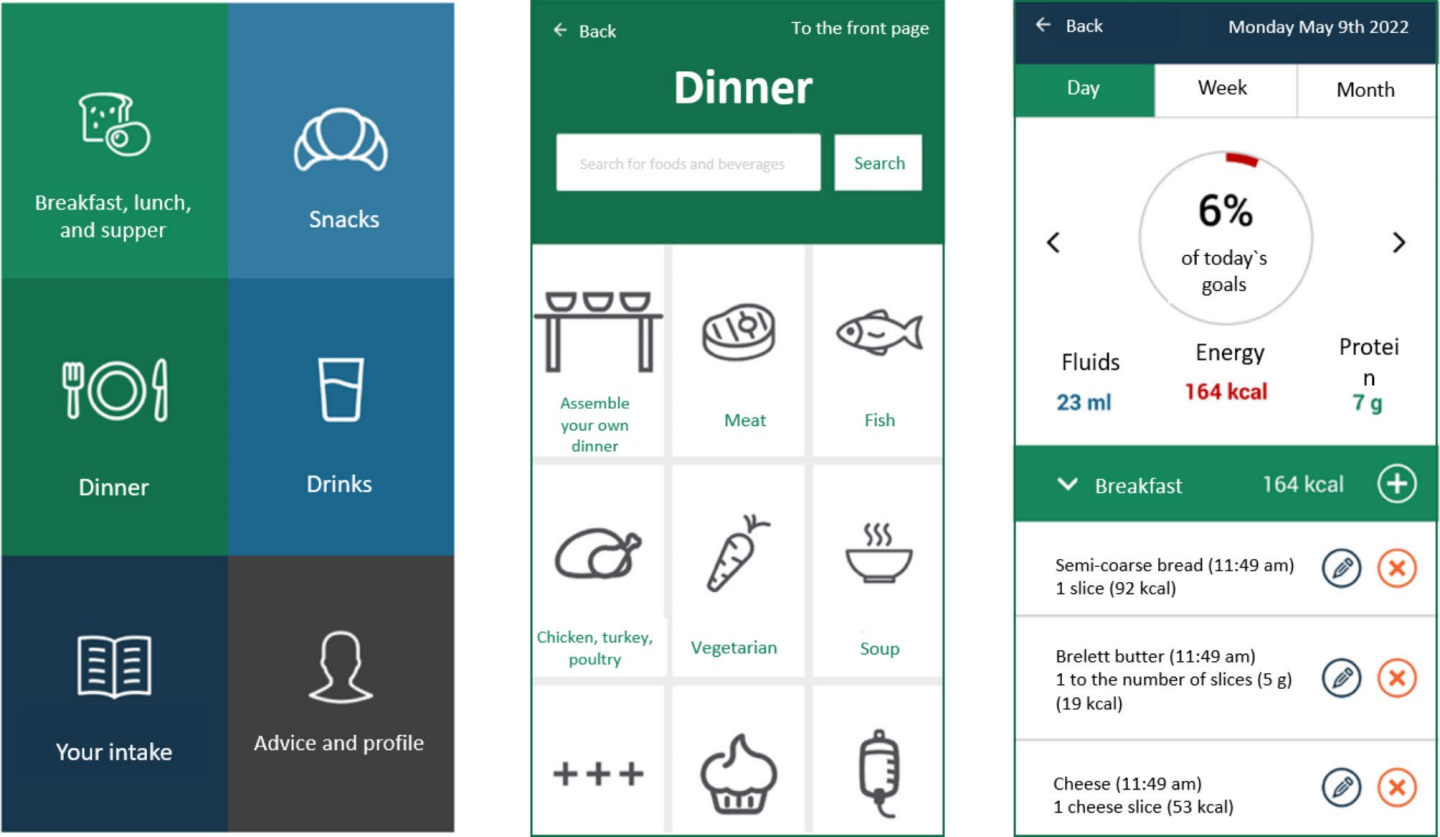
The MyFood app. From the left: (1) Main menu of the dietary recording function; (2) Menu for recording of the dinner meal; (3) Evaluation of recorded intake compared to estimated requirements for energy, protein, and fluid.

The data flow in MyFood uses a web form and secure storage in “Services for sensitive data” (TSD, Tjenester for Sensitive Data) at the University of Oslo and the health care providers had to apply for access and log-in through a secure log-in solution for public services in Norway (i.e., BankID), as described elsewhere [20, 23]. Figure 1 illustrates the dietary recording and evaluation functionalities in the MyFood app.

### Nursing staff participants and training

After initial information meetings between the principal investigator of the study (MMP), the project coordinators (GTK and FS) and each of the leaders of the two units, a plan for implementation of the MyFood system as part of a research study was agreed on. The first step of the implementation was to arrange group sessions. In these sessions, the nursing staff were trained on how to record and monitor the nutritional intake of the participating patients with the MyFood app and how to use the decision support functionality on the MyFood website. The group sessions were conducted physically at the nursing home and digitally at the hospital. Two nursing staff in the nursing home and three in the hospital unit were designated as local project administrators (PAs) and received allocated time for the task. The PAs had the responsibility of recruiting patients and supervising their colleagues. They received special training in how to use MyFood, procedures for recruiting patients and collecting data. The project coordinators (GTK and FS) had the role of train-the-trainers (i.e. the PAs), giving training in MyFood so that they could provide support to their colleagues, reminding them about the project and following up with the participating patients.

### Patient recruitment and nursing staff follow-up

Patients were recruited at admission to the nursing home or the hospital. The patients received oral and written information about the study and signed an informed consent form if they wanted to participate. Patients with a life expectancy of less than 6 months were not eligible for inclusion.

Project tablets (iPad mini 32GB) with the MyFood app installed were available at both locations for use. The nursing staff were instructed to record the participating patients’ intake of foods, beverages and medical nutrition products in the MyFood app for three consecutive days. For patients at risk of malnutrition, defined as a Mini Nutritional Assessment (MNA) score ≤ 11 or a Nutrition Risk Screening (NRS-2002) score ≥ 3, or patients having a dietary intake covering less than 75% of estimated requirements, continued recording in MyFood was recommended. The nursing staff were told that patients able to perform the dietary recording themselves could preferably do so, but that they should record on behalf of patients not able to record e.g. due to impaired cognitive function. For patients at risk of malnutrition or having an insufficient dietary intake (< 75% of requirement), nutritional measures should be initiated. Recommendations for tailored nutritional measures could be obtained from the MyFood web report. They were also instructed on how to use the web report to create a nutrition care plan and to document nutrition in the electronic patient record.

### Data collection and participant characteristics

Seven registered nurses and five nursing assistants (11 women and one man) were included in the interviews. They were between 21 and 54 years old (median 31.5). They had an average of 11 years of clinical experience (range 0.5–33) and on average 7 years (range 0.5–33) of experience in the current unit. The inclusion of participants to the interviews followed a purposive recruitment procedure. This involved the nurse leaders and the project administrators identifying possible respondents who were available on the days the interviews were conducted.

Nursing staff demographics (age, sex) and work characteristics (profession, work experience) were collected before the interview. The third author (GTK) conducted the interviews in the nursing home and the fourth author (FS) conducted the interviews in the hospital. The interviews lasted 17–59 min. The first interview in the nursing home setting served as a pilot interview to test the interview guide, however, as the pilot interview did not lead to any changes in the interview guide, the data were included in the analysis.

The interviews were recorded with a digital voice recorder (Olympus WS-853). Notes were taken immediately after each interview. The audio recordings were transcribed verbatim using the software f4transkript (Marburg).

### The interview guide

A semi-structured interview guide was developed with focus on personal, contextual and intervention-related factors affecting the implementation of MyFood, based on domains and constructs from the CFIR framework [28]. The interview guide included questions about experiences with the use of MyFood, usability, training and available resources, communication, leadership engagement, factors associated with participating in the study, and compatibility of MyFood in the organization.

### Data analysis

The transcripts from the individual interviews were analyzed in a step-wise manner using thematic analysis as described by Braun and Clarke [37]. The software NVivo version 1.6.1 (QSR International) was used to perform the analysis.

First, the transcripts were read by three of the authors (CV, LFA, MMP) to get an overall overview of the material. Second, initial themes and sub-themes were created based on the domains and constructs of the CFIR framework [34]. Third, data were deductively analyzed by the last author MMP into the themes: (1) Intervention characteristics, (2) Context, and (3) Individuals involved. Fourth, the themes were reviewed in several iterations by the first (CV) and the last (MMP) author to establish the meaning and interpret the results. The final step was that the authors discussed and reviewed the themes and sub-themes and subsequently renamed and re-arranged them into a final structure. Due to coinciding results from the nursing home and the hospital locations, respectively, the results from the analysis were merged and presented together rather than separately for each setting.

Trustworthiness in the analysis [38], including credibility, confirmability, dependability and transferability [39], was emphasized. This included involving all authors in the development of the interview guide and audio taping and transcribing the material verbatim. It also included analyzing the data systematically, involving the first (CV), the second (LFA) and the last (MMP) authors in the analysis and the interpretation of the results.

### Ethics

The Regional Committees for Medical and Health Research evaluated this study to be outside the scope of the Norwegian Health Research act. The study was acknowledged by the Norwegian Centre for Research Data (NSD), ref. number 135175. The Chief Information Security Officers in the involved municipality and hospital approved the study. Informed consent was collected from all participating patients and the nursing staff participating in qualitative interviews.

## Results

### Overview

**Fig. 2:**
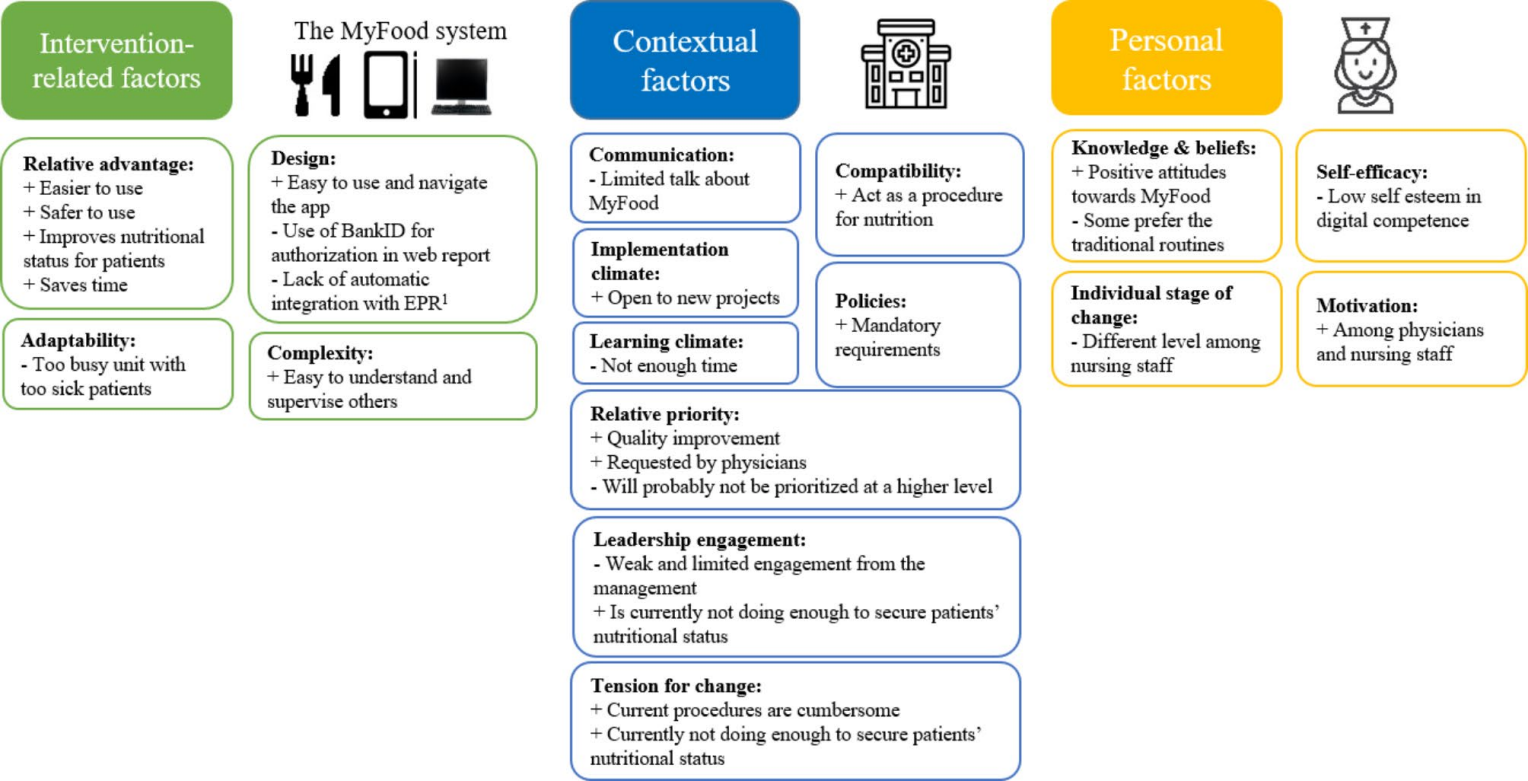
Overview of the main themes (in coloured boxes) and sub-themes (in bold). (The sub-themes correspond to constructs of the Consolidated Framework for Implementation Science [34].) + indicates facilitating factors. – indicates hampering factors. ^1^EPR: Electronic patient record.

The transcripts were analyzed into three main themes covering factors affecting the implementation of MyFood based on the CFIR framework: [1] Intervention-related factors (i.e., CFIR Intervention Characteristics, [2] Contextual factors (i.e., CFIR Inner setting), and [3] Personal factors (CFIR Characteristics of individuals). The main themes and sub-themes are illustrated in Fig. 2.

### Factors related to the MyFood intervention

The intervention in this context was the MyFood system. Most of the nursing staff perceived MyFood as having several advantages compared to the current practice, as described as *Relative advantage* in the CFIR framework [34]. MyFood was perceived as easier and safer to use, and especially the automatic calculation of nutritional intake compared to individual patient requirements was regarded as a huge quality improvement of the nutritional treatment and follow-up. The nursing staff reported becoming more aware of their patients’ nutritional situation, leading to more patients improving their nutritional status.

“*10 times better. […] Safer, more secure system that ensures good nutritional intake for more patients”*. (Registered nurse 4)

Time savings were also perceived as a relative advantage as the majority of the nursing staff experienced saving time using MyFood, compared to the current paper-based procedures.

“*I have actually calculated this. 25 minutes slower with dietary records [current practice]. It is due to the calculation, related to which foods are consumed. […] 25 minutes each shift, this accounts for one hour a day, which is seven hours extra per week.*” (Registered nurse 2).

Regarding *adaptability* and the degree to which MyFood was perceived as able to be adapted, tailored, refined, or reinvented to meet local needs [34], the impression of some of the respondents was that their unit was not the most suitable due to a high diversity of diagnoses and a busy schedule.

*“I think maybe this is the wrong unit to use this [MyFood] because it is very busy and we have so many different diagnoses meaning that some eat a lot and some eat very little. So maybe for another time, a more quiet department where you actually have time and the patients are there over a longer period. Because in our unit, it’s like, they are so sick that there is a reason why they eat little, right”*. (Registered nurse 7)

Some indicated that units with younger patient groups might have been a better implementation arena than the geriatric units involved in the present study.

“*Maybe units with younger patients could use it themselves if they have it on their cellphones, then we would have saved time. […]*”. (Registered nurse 3)

When the informants talked about the *complexity* [34] related to the implementation of MyFood, the general feedback was that the MyFood app was easy to understand and to supervise colleagues in.

Regarding the *design quality and packaging* of MyFood (i.e. how the intervention is bundled, presented, and assembled) [34], the impression was that the functionality for dietary recording and evaluation in the MyFood app was easy to use and navigate. However, the functionality of the MyFood web report, including documentation in the electronic patient record, tailored recommendations for nutritional treatment and a draft for an individual nutrition care plan was perceived as more challenging by some. A specific barrier was the requirement to use BankID (Norway’s secure log-in solution) and that the data was not automatically transferred between different electronic patient record systems. It was regarded as cumbersome that data from MyFood had to be manually transferred to the electronic patient record.

“*We should get it included in our report systems, MyFood should be able to adapt to different report systems in different municipalities – they have to communicate. […]. Even perhaps between the hospital and the municipality*”. (Nursing assistant 1)

Several informants suggested that MyFood would be even more valuable if the system could follow the patient during the institutional stay and in the patient’s home.

“*It can contribute to older people obtaining a better nutritional status throughout the entire, both at home and with us. If it can follow the user. […] If it shall have a purpose, it should be used both at home and at the institution. In municipal services, it should be used both by home care nurses and by us. Because our patients are going home, they are short-term, right. Then it would have been completely perfect. Then we can distribute the report when someone is admitted or discharged. I think that if this is going to be really good for patients at short-term departments, the home care services should also use it [MyFood]*”. (Nursing assistant 1)

A possible value for the patient’s next-of-kin was also suggested.

“*[…] It will not be a question when someone is confronting you with: “mother has not eaten, she has lost weight”. “No, look here, she has not”. You can use that physical thing [the MyFood app] and show – “look, she has gained weight. Eaten this amount yesterday, and this amount the day before that. Here is her weight curve”. It would have been absolutely fantastic, and aid in everyday work life, I’m just saying. You have more to show, to the next-of-kin*”. (Registered nurse 4)

In summary, the suggested improvements of the MyFood system by the nursing staff were to integrate the system with existing electronic patient systems, to use MyFood throughout the patient course and to involve the patient’s next-of-kin.

### Factors related to the context

Factors related to the context are divided into the inner setting (factors inside the organization) and the outer setting (factors outside the organization) [34]. In the following paragraphs, the inner contextual factors are presented before the outer contextual factors.

Concerning the *networks and communications* in the organizations involved [34], the interviews revealed that structured arenas for information sharing and dialogue in terms of arranging regular meetings and using email as an information channel existed. However, the nursing staff said that they did not have much dialogue about the MyFood intervention through these channels.

“*We have not talked a lot about it, to be completely honest. From what I have seen. It’s more like, you do the recording [of food intake in MyFood], but in busy everyday life, it’s not much talk about MyFood. […]”*. (Registered nurse 2)

Regarding *Leadership engagement* [34], the results indicated that leadership engagement was weak and that leaders only to a limited degree were closely involved.

“*They [the leaders] have not involved much. They kind of threw it at us. Like everything else. […] I think it would have helped if they engaged a little more about things that are newly implemented. In general. They should show their heads a little more frequently*”. (Nursing assistant 4)

The informants did not experience that the implementation of MyFood was communicated from the management, nor did they talk with their leader about it.

“*Not a lot [talked to the managers about MyFood], other than she sent a few emails about how to log in and do this and that. Not much talk other than that*”. (Registered nurse 3)

Communication from the management through e-mails was not a preferred communication method because the nursing staff were concerned that information via email did not reach all employees, and that they would like to have the opportunity to discuss the use of MyFood with their colleagues more than just receiving written information.

“*Not everyone checks their job emails every day. You do not have time for that. And not everyone wishes to read their job email from home. […] I think it could have been done a little differently to introduce it in the beginning, rather than take all information through email. Because it is not the same as face-to-face in a morning meeting*”. (Registered nurse 4)

However, MyFood was discussed to a very limited extent in staff meetings, and only in the form of information and reminders. The nursing staff wanted the leaders to initiate nursing-related professional discussions about MyFood.

“*At least it’s on the agenda, but I don’t know who brought it up. I haven’t been there. So I don’t think it will be brought up either. It is there, on the agenda. It says: “remember MyFood”. So then the question is sort of how it came about, I think it comes back to the leadership”.* (Registered nurse 2)

When talking about *Implementation climate*, in terms of the absorptive capacity and organizational support for change [34], the nursing staff perceived their department as being open to new projects.

“*I experience that our department is often participating in projects and that we are a department where professional knowledge is quite high among all, everyone is equally hungry for things”*. (Registered nurse 5).

The informants in the study experienced what CFIR call a *tension for change* which implies that they perceived the current situation as intolerable or needing change [34], based on telling that the current procedures with paper-based dietary recording were perceived as of poor quality, cumbersome and old-fashioned.

“*You have to go up to the kitchen, check how many calories, and if a patient has a special diet it is something different, you have to call the kitchen, and that takes much longer time. Like everything. And I’m not very good in math, so it’s like, you have to use a calculator and… no. It takes a long time to record [dietary intake] the way we do things now”*. (Nursing assistant 1)

Some nursing staff experienced that their department did not do enough to secure good nutritional status among the patients and believed that MyFood could improve this situation.

“*And I really think nutrition is so very important. We have a lot of deviations in nutrition in the department. Very serious deviations, so we could have prioritized differently. That’s my personal opinion”* (Registered nurse 2).

When asked about *Compatibility* (i.e. the fit between MyFood and the individuals’ values as well as existing workflows and systems) [34], several informants emphasized the potential of MyFood, and one of them also compared it with a procedure.

“*I think this [MyFood] has great potential. Because it will be similar to a wound procedure. You follow it, and then you change it if the wound change, right. […] It [MyFood] becomes a practical tool that actually gains the patient and us. That is why this is so great, yes. I think»* (Nursing assistant 1).

*Relative priority* is the individuals’ shared perception of the importance of the implementation within the organization [34]. Several informants believed that implementing MyFood would lead to an easier workday, save time and improve quality in nutrition-related work.

“*I think the utility is obvious compared to what we can obtain, that it becomes much more visible, right. Now, we for example complete dietary records, daily dietary records. On paper[…] There are a lot of lists, helter-skelter. It is difficult to go back to get an overview. […] With this system [MyFood] it’s all here. And the physician can go into the app and obtain the information, very easy”*. (Nursing assistant 1)

Many of the nursing staff said that the physicians had requested the MyFood assessment reports and that the physicians had appreciated the overview of the patients’ nutritional status provided via MyFood.

“*The physician was very, like, “here you could have used MyFood”. The physicians have been very active. At least for some of the patients who they feel that it could be useful*”. (Registered nurse 6)

There was an impression that from a longer time perspective the municipality would not take the costs of implementing such a tool, and this may be related to the relative priority on a higher level.

“*I think a lot of people think it’s a bit like that, this place isn’t going to spend money on implementing such a tool, so why do we have to do it? […] The tool is great […] I just think that they feel that they are in a study, and are not allowed to use it later”.* (Registered nurse 4)

A factor in the *learning climate* construct is that there is sufficient time and space for reflective thinking and evaluation [34]. An experience of not having enough time was mentioned.

“*I wish we had more time in advance. A workshop where we could sit and work and look at it […] Got practice[…]. Because it gets a bit like every now and then*”. (Nursing assistant 1)

When it comes to the nursing staff’s recognition of the *patients’ needs and resources* [34], the interviews showed that the patients did not use MyFood themselves, but that the nursing staff used MyFood on behalf of the patients. There was a perception that elderly patients would not be able to use MyFood themselves. The nursing staff seemed to be most concerned about their time use and effectiveness, rather than the patient perspective.

“*When I come into [the room of] a 100-year-old lady, she doesn’t understand it completely. She might not understand, like, she has maybe never seen an iPad before. However, I did explain, that I should record food and beverage intake. And then she said, “that is completely fine”, that is what most people say*”. (Nursing assistant 4)

Mandatory requirements related to national guidelines for the prevention and treatment of malnutrition [40] were emphasized as important concerning *external policy and incentives* [34].

“*But we are required by law to document everything they eat, and nutrition is very important and very much in focus. So MyFood is an aid that I absolutely think is very good for us*”. (Registered nurse 5)

### Factors related to personal characteristics

Regarding the nursing staff’s *knowledge and beliefs* about MyFood in terms of their attitudes toward and the value placed on the intervention[34], some of the informants had very positive attitudes towards the MyFood system and the project.

“*I am very positive about it. […] I think it is easier to use this app. […] I am very much in favour of us operating MyFood. That’s the next step. […] We have gotten a better overview through MyFood, it’s more efficient use versus food lists [paper-based food records]*”. (Registered nurse 2)

However, others were more skeptical.

“*But some people are not very satisfied. They think it’s all right with the list on the wall [current practice with paper-based forms]. […] It was kind of when the app was down [technical bugs] there was much negativity*”. (Nursing assistant 5)

When talking about *Self-efficacy* and the individual’s belief in their capabilities to use MyFood [34], some of the nursing staff had low self-esteem in their abilities to use a digital tool for nutritional care.

“*When it comes to using it I have some challenges. Because I am not technical. But otherwise, things are going very well. […]. It is not the app’s fault that I am slow. I’m not so good with such apps, I don’t use them much myself. […]. I think it’s me that is worried about doing mistakes, so I don’t dare to do it alone without colleges that can do it*”. (Nursing assistant 1)

The nursing staff were at different levels related to their *individual stage of change* [34]. Some were very positive to change and testing new tools.

“*Positive. Fun! I think things like this are exciting! Click, learn and explore*”. (Nursing assistant 3)

Others of the nursing staff were at a later individual stage of change.

“*Maybe some of the old trotters are somewhat slow. They are everywhere in all workplaces*”. (Nursing assistant 4)

The nursing staff seemed to think the MyFood app was easy to use. However, the majority did only use the app and not the MyFood web report, which indicates that the implementation was still in an early phase where the intervention was only partially adopted.

“*How I should go into that web report and stuff like that, I didn’t prioritize to go in there. Because I didn’t have time. But to use the app and such, the information has been OK. But the other [web report], I didn’t*…”. (Registered nurse 7)

*Motivation* [34] was mentioned as an important factor, both among the nursing staff and the physicians.

“*I think they [the physicians] have been very motivated. Because they see that we [the nursing staff] use MyFood, that we are a bit motivated for that. So it becomes a little easier for them to assess the nutrition. Much easier for them. Because we have had many deviations on it, it has been a problem. […] It has been good that the physicians are with us”*. (Registered nurse 1)

## Discussion

The present study found that nursing staff experienced several advantages with implementing a digital decision support system for the prevention and treatment of malnutrition, including quality improvement and time savings in the nutritional follow-up of patients. However, the leadership engagement was low, several nursing staff experienced low self-efficacy in digital competence and they were at different levels of change. Suggestions for how the implementation of MyFood could improve in the future included increasing the level of training, using MyFood throughout the patient course and involving the patient’s next-of-kin.

### The MyFood intervention

Several intervention-related factors were found to affect the implementation. As the MyFood system included two related but not dependent modules, the MyFood app and the website with report, the results indicated that only the MyFood app was fully implemented, whereas the MyFood website with report was implemented only to a limited degree. The interviews provided no explanation as to why the MyFood website was only partially implemented. However, it is not unexpected that complex interventions must be implemented step by step [41], and that the implementation had thus not been fully completed at the time of the interviews.

The MyFood app was experienced as easy to use and navigate, improving the nutritional status of the patients and saving time for the nursing staff. Hampering factors were found to be the log-in solution at the website for the report and the lack of automatic integration between MyFood and the electronic patient record. These facilitating and hampering factors correspond with previous findings when implementing MyFood in specialized hospital wards [21, 22].

Suggestions for how the use of MyFood could add increased value for both healthcare workers and the patients were to use MyFood throughout the patient course. It was suggested that MyFood can be offered both when the patient is admitted to a nursing home and afterwards when the patient is transferred to home and followed up by the home care services. It was also suggested that the patient’s next-of-kins could be involved as important stakeholders in the use and follow-up of MyFood. A Norwegian study about implementing digital technology among elderly people with dementia in residential care facilities concluded that next-of-kins are highly salient in this context [42]. However, the study also showed that while some next-of-kin have resources and can be highly engaged, others are unable or unwilling to be active participants in their family members’ lives [42].

None of the patients included in the present study used the MyFood app themselves, but the nursing staff used MyFood on behalf of the patients. The nursing staff did not believe that any of the included patients would manage to record their dietary intake in the MyFood app. Previous findings showed that patients who used MyFood became more aware of their nutritional requirements and more motivated to eat to reach their daily nutritional targets, however, these patients had a lower mean age [21].

### Leadership engagement

The leadership engagement was reported to be weak and limited and the nursing staff experienced that the management was currently not doing enough to secure patients’ nutritional status. Findings from the current study showed that the nurse managers only to a limited degree were active supporters of the use of MyFood. This is in line with a systematic review by Gifford et al. [30] that found that when the employees were dissatisfied with the support from their leaders, this acted as a barrier to implementation.

Systematic reviews have found that strong leadership is essential to obtain effective nursing practice in supporting patients’ nutrition [2, 26]. Others have also highlighted the vital role of nurse managers in the implementation of evidence-informed practices, including providing a supportive culture and environment [27–33]. Important leadership qualities to support implementation such as proactive, supportive, knowledgeable, perseverant, relation-oriented, functional, and strategic, as described by Castiglione [28] were reported to be almost absent among the leaders in the current study. The limited active support from the leaders may contribute to the explanation of why the implementation of MyFood was not fully successful. As suggested by the nursing staff in the current study, the use of MyFood should be requested to a larger extent by the leaders and also be addressed in staff meetings and shift handovers.

### Formal and informal implementation support

In the current study, project administrators were dedicated as implementation supporters to provide learning and follow-up of MyFood and to motivate the nursing staff in using MyFood. A recent review pointed to implementation support as a frequently used approach to strengthening implementation processes by supporting and assisting healthcare providers in their use of new interventions [43]. The review highlighted that implementation support practitioners, (such as the project administrators in the current study) can only reach their full potential when there is established a trusting relationship between them, the staff and the leaders [43]. As the leaders in the current study were not so dedicated, this may have affected the project administrators and made their tasks as implementation supporters difficult.

MyFood received unexpected implementation support from the physicians. The nursing staff reported that the physicians were motivated to use MyFood, and requested the MyFood assessment reports. Several nursing staff said that the physicians had appreciated the overview of the patient’s nutritional status provided by MyFood. This is in line with previous results from a pre-implementation study assessing potential barriers and facilitators for use of MyFood in two specialized hospital wards [22], which found that nursing staff believed that the physicians would probably trust MyFood more than the current practice with paper-based dietary records. However, prejudices among some physicians regarding the role of nutrition in the treatment process were also identified [22]. Eide et al. [12] found that nurses were frustrated about the physicians’ low involvement and engagement in the nutritional care of the patients and that support from physicians in nutritional care made it easier to prioritize nutrition.

### Self-efficacy and individual phase of change among the nursing staff

The present study showed that the nursing staff had varying levels of digital competence, defined as “the set of knowledge, skills, attitudes, abilities, strategies and awareness that is required when using information and communication technology (ICT) and digital media” [44]. A systematic review of health care professionals’ competence in digitalization [45] described that key competence areas are knowledge of digital technology and digital skills required to provide good patient care. Health care professionals’ attitudes and experiences influence their willingness and motivation to use technology [45]. This is in line with the finding in the present study of the nursing staff being at different levels related to their individual stage of change. Some were very positive to change and testing new tools, whereas others were more resistant to change and skeptical. The review also highlighted that organizational and collegial support is required for the effective adoption and use of new technology [45].

A tendency to resignation among some of the nursing staff was also found in the present study. As they experienced some of the patients being very ill with several diagnoses, they perceived that the patient would not eat anyway so there was no point trying. This finding is worrying, due to mandatory requirements to provide nutritional assessment and treatment for those identified to be at risk of malnutrition [40].

### Coinciding challenges in hospitals and nursing homes

As the study was conducted in two different locations, one could expect that the results would differ due to specific location-related factors, however, we found that the results were coinciding. This may be due to several barriers related to the current treatment and follow-up of malnutrition previously demonstrated in both nursing homes [33] and hospitals [12] including lack of focus, poor routines and limited knowledge on nutritional treatment among nursing staff [12, 33].

### Strengths and limitations

There are several limitations to this study. The study included a limited number of nursing staff. However, the data were rich enough to provide insight into the implementation of MyFood from the nursing staff’s perspective and included staff from both hospital and nursing home.

As the results revealed that leadership support was weak, the involvement of leaders in the interviews could have provided important contributions and strengthened the study. All but one of the nursing staff participating in the interviews were female, which means that we were not able to capture any potential skewness related to genders in the experiences with the implementation.

A strength of this study is the use of an established framework in implementation science to understand, describe and identify factors that affected the implementation. After the analysis process, an updated version of the CFIR was published [46]. After reviewing the updated CFIR, we do not believe that this update would have affected the results of the present study. Although there are several updates and relocations in domains and constructs, these can be mapped back to the original domains and constructs [46]. Another strength is emphasising trustworthiness in the analysis [38], including credibility, confirmability, and dependability [39].

## Conclusion

This study showed that nursing staff experienced several advantages with implementing a digital decision support system for the prevention and treatment of malnutrition, including quality improvements and time savings in the nutritional follow-up of elderly patients. The results indicate that the leadership engagement was weak and that some nursing staff experienced low self-efficacy in digital competence. Suggestions for how the implementation of MyFood and similar interventions can improve in the future include increasing the level of training, using MyFood throughout the patient course and involving the patient’s next-of-kin.

## Data Availability

The datasets used and/or analysed during the current study are available from the corresponding author on reasonable request.

## Abbreviations

CFIR: Consolidated Framework for Implementation Research
EPR: Electronic patient record
MNA: Mini Nutritional Assessment
NRS: Nutrition Risk Screening
NSD: Norwegian Centre for Research Data
PA: Project administrator
TSD: Tjenester for sensitive data (Services dor sensitive data)
